# Investigating the citing communities around three leading health-system frameworks

**DOI:** 10.1186/s12961-023-01075-6

**Published:** 2024-01-26

**Authors:** George Weisz, Jonathan Harper

**Affiliations:** Dept. Social Studies of Medicine, McGill University, 3647 Peel Street, Montreal, QC H3A 1X1 Canada

**Keywords:** Citation community, World Health Organization, Alliance for Health Policy and Systems Research, Harvard-World Bank Flagship Program, Global Burden of Disease, Health Policy and Systems Research

## Abstract

Of numerous proposed frameworks for analyzing and impacting health systems, three stand out for the large number of publications that cite them and for their links to influential international institutions: Murray and Frenk (Bull World Health Organ 78:717–31, 2000) connected initially to the World Health Organization (WHO) and then to the Global Burden of Disease Project; Roberts et al. (Getting health reform right: a guide to improving performance and equity, Oxford University Press, Oxford, 2004) sponsored by the World Bank/Harvard Flagship Program; and de Savigny and Adam (Systems thinking for health systems strengthening, WHO, 2009) linked to the WHO and the Alliance for Health Policy and Systems Research. In this paper, we examine the citation communities that form around these works to better understand the underlying logic of these citation grouping as well as the dynamics of Global Health research on health systems. We conclude that these groupings are largely independent of one another, reflecting a range of factors including the goals of each framework and the problems that it was meant to explore, the prestige and authority of institutions and individuals associated with these frameworks, and the intellectual and geographic proximity of the citing researchers to each other and to the framework authors.

## Introduction

Citations have many functions, as a lively analytical literature attests (e.g., [3, 20, 28, 43]). A citation may express perceived intellectual debt, provide intellectual authority or legitimacy for arguments and claims, offer a target for criticism, be part of a literature review, point to further relevant information, or simply serve as proof that one is familiar with the literature (or at least the work of potential reviewers). They can also serve as a form of self-presentation used to directly position work within scientific traditions or schools of thought. The pattern of use varies from discipline to discipline and from group to group [11, 27, 46]. In what follows, however, we will not be concerned with *how* citations are used but rather with the citing publications themselves and what Hansen [19] has termed “the citing community”. The arrival of citation indexes and databases has added new dimensions to the study of citations [14, 24], so that close examination of such communities can now expand our understanding of knowledge dynamics in the domains examined (e.g. [35]). Much has been learned from scientometric studies of large publication corpuses, the life cycles of highly cited papers, and the factors behind these trajectories (e.g. [9, 10, 18]). There is however little if any discussion in the literature of what citation collections that form around a particular publication represent or what holds them together. Are they simply fleeting aggregates based on momentary interest in a single text, or do they represent more stable and long-lasting communities of scholarly interest? This paper is thus an exploratory effort to understand several aspects of a ubiquitous product of scientific research applied to the domain of Health Policy and Systems Research (HPSR). We begin with the idea that a corpus of citations around a work can be treated as a distinctive citation community (momentary or lasting) that can be analyzed along different axes to better understand the fields of studies of which it is a part. We go on to a case study that examines the citation communities around three highly cited and institutionally linked works offering frameworks for studying health systems within the world of Global Health and particularly the emerging field of HPSR.

## What are health systems?

Research on health systems emerged in international health and within the World Health Organization (WHO) during the 1960s and 1970s as an instrument for rationally planning its healthcare activities and those of member states [16]. It remained marginal at the WHO, receiving little funding in comparison to disease-based biomedical research [49]. During the 1990s, however, growing commitment to expand research to improve health worldwide led to an emergent consensus about the need for such an applied policy field and to the creation in 1998 of the Alliance for Health Policy and Systems Research (AHPSR) which expanded the field beyond the WHO while remaining close to that organization. In 1998 as well, the European Observatory on Health Systems and Policies was established by the WHO Regional Office for Europe. The WHO’s turn-of-the-millennium *World Health Report (WHR 2000)* [51] analyzed and ranked health systems worldwide and provoked immense controversy while increasing the visibility of and focus on health systems [30]. In the context of rapid multiplication of actors and financial resources in global health, concern with health systems intensified in the following years. This concern was based on the belief that too many resources were being invested in narrow disease-based programs that failed because health systems were unequipped to implement them. The widespread conclusion was that greater investment in “health systems strengthening” (HSS) was needed [17]. There was little consensus about what precisely HSS meant or how to achieve it [42, 55]. Some critics went so far as to argue that for many institutions, HSS was a rhetorical device [29]. But such disagreements did not make the term less relevant and indeed underscored the need for more rigorous research.

Some formulations or definitions of health systems, like the popular one proposed by the *WHR2000*, simply sees them as the sum of institutions and people that affect the health of individuals and populations. This leaves open a wide array of possibilities for defining relevant components. It is also not clear just how such huge aggregates of institutions and people constitutes a “system.” They are certainly not ordered structures designed rationally to serve specific ends; the reality is that health systems have grown haphazardly and by accretion. The term “system” thus has had two distinct meanings beyond simple collections of institutions and people: 1) the functional logic that an analyst can detect beneath apparent institutional chaos; 2) the ambition to make healthcare institutions behave like rational systems pursuing stated goals in the most efficient way possible, however efficiency is defined. Governments have pursued goal 2 since long before HSS became both a catchphrase and genuine aspiration. Nonetheless this objective provides perhaps the simplest and most accurate definition of Health Policy and Systems Research.

Strengthening health systems, the foundational goal of HPSR, requires working conceptual frameworks for analyzing their structure and functioning. Such frameworks preceded the institutionalization of HPSR in the twentieth century. Early formulations ranged from the Donabedian [13] model of health services linking input processes and outcomes to Roemer’s typology of health systems based on levels of government control [38]. Since then, numerous frameworks of this sort have been proposed. A survey published in 2012 found 41 different frameworks [22] while another [26] found 49. Neither list claims to be exhaustive and, as we shall see, meanings of “frameworks” can be fluid. Frameworks can have different functions including understanding, comparing, and evaluating systems, as well as informing or even suggesting protocols for change. They can also be distinguished by scope, applying to systems as a whole, to specific sub-domains like maternal and child health, to major system functions like human resources or governance, and to the relation of health systems to other societal sectors. They can also reflect different values and goals (cost-effective efficiency versus equity for instance). Alternatively, their high level of abstraction may also permit them to co-exist with quite different values and goals without being based on or tied to any of them. This makes efforts at synthesis, according to some, both impossible and undesirable [45]. For others, however, this plethora can seem troubling and confusing, resulting in periodic calls for and efforts at framework convergence or synthesis (e.g. [26, 34, 40]). Most studies of frameworks focus on explaining their similarities, differences, and/or inadequacies. In this paper, however, we propose to examine how a few of the most popular ones are utilized.

## Methodology

Word searches for terms like “system frameworks” in databases yield huge numbers of hits, with the vast majority having little to do with system frameworks, Global Health or HPSR. We thus chose another strategy. Based on the surveys and discussions mentioned above, we collected the articles discussed, relevant work they cited, and later works which cited them. We then chose those publications that actually present “frameworks” and then further selected those covering entire health systems rather than individual components or functions. We ended up with 12 publications out of the 65 we originally found. As a proxy for popularity and acceptance, we focused on the number of times framework articles have been cited. Of the many databases available, we chose Google Scholar despite recognized quality control issues [2], for several reasons. First, other databases like Scopus and Web of Science focus on periodical literature and are weak on books and institutional reports, Consequently, getting even less than complete citation data for these can take several steps, as we will see below. Google Scholar, in contrast, includes a wider range of publications meaning that they can better capture framework publications promoted by international institutions published in the form of books and reports. Second, while small differences in numbers of citations are not valid using this source, there is no reason to discount the very large differences of scale that turned out to be the case.

Of the publications we found, most had been cited only a handful of times according to Google Scholar as of early November 2021. There were two exceptions.; Several had been cited a little over 100 times: [5] (114), [31] (119) and [44] (119). There was then a huge jump, with four publications cited over 1000 times: [33] (1370), [12] (1131), [37] (1113) and [25] (1227). Significantly all four were promoted at some point by major international organizations or commissions. Three of these (the Kruk article was published too late) are among the 5 highlighted in the most recent survey of frameworks [26]. The difference between these and the first group of publications was far too great (a ratio of nearly 10 to 1) to be attributed to lack of quality control in the database. Furthermore, while other databases had fewer hits for all publications, they mirrored the discrepancies between the highly cited works and the rest. We thus felt comfortable focusing on these four publications which had the added benefit of providing a large number of citations for analysis.

However, further research showed that one of the highly cited works, Kruk 2018, was problematic. Produced by The Lancet Global Health Commission, it promoted a new framework aiming to make “quality” a central goal of HSS. This was part of a wide-ranging campaign to improve the quality of health systems and was accompanied by the Bellagio Declaration on high-quality health systems signed by 19 organizations and national ministries. This sudden (re)discovery of quality certainly deserves a study of its own, but its significance had little to do with the system framework that was proposed. Of the 100 most cited works citing this article, only five mentioned the framework in the text and two of these were by Kruk herself. While it is common for framework articles to be cited without any discussion of the framework (see below), we decided that this case was too extreme to contribute to our understanding of frameworks and their uses.

This left us with three very highly cited texts. Works citing these texts were thus collected from the Scopus database which, while imperfect, has fewer issues than Google Scholar and which contains valuable metadata. (We chose it over Web of Science which also has metadata, but which is less complete since it waits several years before deciding to cover new publications.) We should point out that it was somewhat difficult to collect all citations relating to [12, 37] because Scopus (like Web of Science) does not handle monographs and organizational publications easily. We searched for titles and lead authors' surnames of the three texts, used the “view cited by” function to download citations for Documents and then Secondary Documents and exported each into Excel files that were combined into a composite Excel file for each framework publication. We examined these lists of citing publications for redundancies and removed duplicate entries. The articles were then read to eliminate publications that did not in fact cite any framework publications. Some queries (annual number of citations, citing journals, institutional and national affiliation of lead authors) simply involved counting information included in the citing publications or the database. In the case of thematic content of citing articles, the two authors separately read, coded the publications and, where necessary, reconciled differences. While counting institutions, journals, and annual number of citations was straightforward, attributing themes is always a somewhat subjective exercise, we fully concede, no matter how many evaluators are involved. Consequently, results can never be truly definitive, and we do not present them as such. More generally, the data shows interesting patterns but is itself incapable of explaining them. Most explanations we propose are thus speculative and based on our understanding of the field. We thus present our results as suggestive, as open to different interpretations, and as a stimulus to further research.

Of the three highly cited works, two are currently supported by major institutions: [37] is the primary source for the “control knobs” framework of the Harvard-World Bank Flagship Program; [12] is the most complete incarnation of the “building blocks” framework promoted by WHO and the AHPSR. Both have thus been chosen for detailed analysis. A third highly cited paper, [33], initially enjoyed the institutional backing of the WHO and provided the framework for World Health Report published in 2000 (WHR 2000), which received enormous attention and criticism for the way it quantified and ranked national healthcare systems [51]. In the wake of such controversy, and a change of leadership in 2003, the WHO abandoned this framework [45] and eventually settled on the building blocks framework mentioned above. Nonetheless, because it continues to be cited with such regularity, and because its co-authors are closely associated with the Global Burden of Disease Project, we decided to include the Murray and Frenk article in this study.

## The three framework texts

In 1997, the World Bank Institute collaborated with Harvard’s T.H. Chan School of Public Health to launch the Flagship Program, an annual fee-based course aimed at building capacity for health sector strengthening through systems level approaches. National and regional courses were soon added. Between 1997 and 2008, the Flagship Program organized 319 short-term training events for more than 19 400 policymakers, analysts, and implementers in 51 client countries [6, 41]. By 2022, the program information asserted that 36 600 individuals from 70 countries had participated in its courses [15]. A system framework was developed from course materials produced in several drafts. In 2003, Harvard economist William Hsiao wrote a paper introducing his 5 “control knobs” [23]. The explicit goal of this framework was system change. The assumption was that acting correctly on any of these strategic knobs would have positive effects on the entire system. Factors like culture, which change only slowly were unequivocally excluded from the model. A year later, the Framework team led by Roberts et al. [37], and including Hsiao, published a book, *Getting Health Reform Right* (*GHRR),* which became the basic textbook of the Flagship program. (It was republished in 2008 and in a 2019 anniversary edition.) It incorporated the five control knobs (Table 1) but also insisted on the importance of political context and the need to make inevitable ethical choices, likely due to the influence of Roberts and political scientist Michael Reich, who co-wrote the chapters on ethics and politics (Roberts et al. 2008 preface). It is worth noting that while this framework had the brand of two powerful institutions, it was marginal to each and largely self-funded. Several studies indicate that World Bank health systems analyses use a large variety of frameworks [8, 48]. One would thus not expect *GHRR* to be as widely cited as other popular frameworks. This is particularly true since its primary goal is system reform; publication by academics is secondary to training policymakers to plan and implement reform. Course fees would presumably further encourage participation by students with some financial—often government—backing, like administrators. In recent years, things have evolved. Academic work is published in a journal associated with this group—*Health Systems & Reform—*edited by one of work’s authors, Michael Reich. This journal publishes a good proportion of the work citing *GHRR*, as we shall see. The Program has recently eliminated fees, increased course offerings, and added online self-learning modules [15], which should in coming years, increase its visibility.

**Table 1:** Most cited health system frameworks

World Health Report 2000 Murray + Frenk 2000	World Bank-Harvard 5 control knobs framework 2003 GHRR	WHO building blocks framework 2007, 2009) STHSS
**Functions** Service provision Resource generation Financing Stewardship **Goals** Responsiveness Health Fair financial contribution	**Knobs** Financing Payment Organization Regulation Persuasion **Performance goals** Efficiency Quality Access **Target popl**.goals Health status Citizens’ satisfaction Risk Protection	**Blocks** Service delivery Health workforce Information systems Medical products Financing Leadership and governance **Intermediate goals** Access coverage Quality-safety **Goals** Improved health Responsiveness Financial risk protection Improved efficiency

**Fig. 1:**
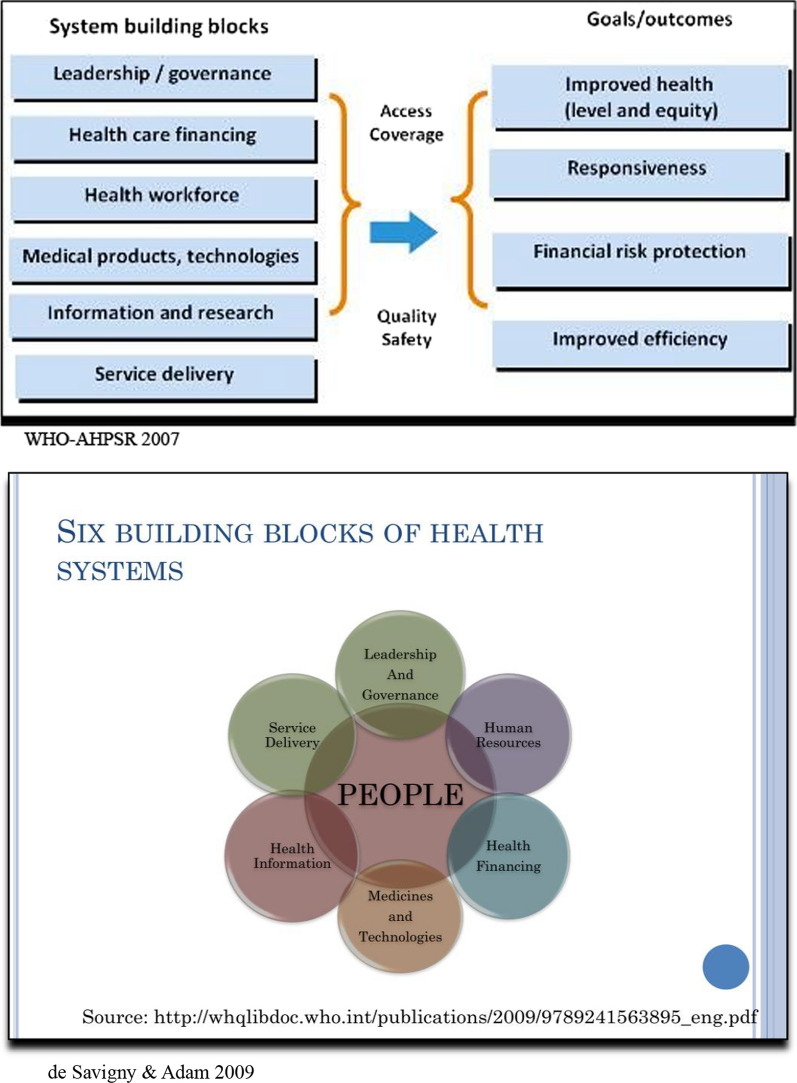
Two versions of building blocks. Reproduced from World Health Organization. (2007). Everybody’s Business: Strengthening Health Systems to Improve Health Outcomes: WHO’s framework for action. P. 3. Reproduced from Savigny, Donald de, Adam, Taghreed, Alliance for Health Policy and Systems Research & World Health Organization. (2009). Systems thinking for health systems strengthening. World Health Organization. P. 32

In contrast, the “building blocks” have been promoted and funded by two influential institutions, the WHO, and its affiliate, the AHPSR. It was taken up almost immediately and with slight variation by numerous global health agencies [17, 47]. The initial statement was published by WHO and AHPSR in 2007 and introduced the six “building blocks” as the basic structural units of health systems [52]. But to many it appeared that the blocks functioned as isolated silos with little regard for their systemic interconnections or the importance of people. Consequently, 2 years later, the two organizations produced a second publication, *Systems thinking for health systems strengthening* (*STHSS*) [12], that sought to fill both lacunae. System thinking was added as a central element and its importance was discussed at considerable length (in contrast to the “control knobs” whose systemic impact was largely taken for granted). One change had to do with replacing a linear representative image, like those used by many other groups, with one emphasising systemic links (Fig. 1). Nonetheless, changing images is easier than changing practices. Building blocks do not necessarily lead to systemic research, a fact that is regularly noted in the literature (e.g. [1, 4]). This framework has been criticized by leaders of the Flagship program for being essentially a checklist of WHO areas of interest rather than a framework for reform [36]. Others have complained that it is too static and ignores systemic impacts and long-term outcomes [32]. Several “dynamic” frameworks have been proposed for entire systems [44, 45] and subsystems like neonatal health [39]. Nonetheless, the WHO building blocks, supported by several international and national organizations, and promoted in numerous articles, reports, and tutorials, are generally considered to have become the most widely used framework for HPSR. Several major Global Health funders like the Global Fund, GAVI and PEPFAR channel funds for health system strengthening based on this framework [47]. It is precisely their descriptive character that makes them so useful for the eclectic mixture of academics and policymakers that make up the growing health systems research community. The blocks define health systems in a broad and inclusive way that allows large numbers of authors and institutions to position their work within its categories.

The article by Murray and Frenk [33] provided the framework for the widely cited but controversial WHR 2000 which, as noted above, ranked the performance of national health systems. Although the controversy and criticism it provoked led the WHO to abandon it after 2003, this attention is undoubtedly one reason why the article, like WHR 2000, continues to be frequently cited even though it is the only one of the three frameworks that is not actively promoted by a major institution. There are other reasons as well for the sustained interest this paper generates. The authors, Chris Murray and Julio Frenk, are among the most prolific authors in the global health world and they frequently cite their own article. They are responsible, individually or together, for 25 of the 451 articles listed in Scopus as citing this paper. More important they are influential figures in Global Health and have co-authored papers with numerous individuals, particularly but not exclusively in the world around the Global Burden of Disease Project (GBD) financially supported by the Bill and Melinda Gates Foundation. Authors within or close to this sphere of intellectual influence are thus likely to be familiar with this seminal article. Furthermore, one purpose of this framework, evaluating health systems, remains a popular concern within Global Health^1^ as does another of its key points, the importance of responsiveness to the needs and desires of people who use the system.

Looking more closely at the frameworks themselves (Table 1) we see that they are broadly similar but with important distinctions. All three include financing as a major structural component. Choosing different terms, all three cover the setting of overall system direction (stewardship, regulation, leadership, and governance). As well, they all consider system responsiveness and better health as major goals. Murray and Frenk is the simplest with only 4 functions and 3 goals suitable for measurable assessment. Unlike the other two, it includes a category for non-financial “resource generation” where resources might be human, physical (facilities and equipment) or knowledge produced by organizations like “universities and other educational institutions, research centres, and companies producing specific technologies … products, devices and equipment” [33, 725]. It is also the only framework that does not list “efficiency” as a goal. Since this last term appears 17 times in the article, this means that efficiency like quality is viewed as a function of “composite goal performance” (721). GHRR is also unique in several respects. There are two categories related to economic issues (payment, finance) and, a category called “persuasion” that reflects its aim to promote change and the acknowledgement that this requires political action to mobilize opinion. It is also the only framework that lacks a category covering health service delivery or provision, because change here is viewed as a consequence of more fundamental changes in the control knobs. STHSS goes into greatest detail by identifying specific areas like health workforce, information systems, and medical products. GHRR does not go into great detail about what constitutes system research, while STHSS devotes considerable energy to justifying, promoting and seeking to improve systemic methodologies.

## Results

**Fig. 2:**
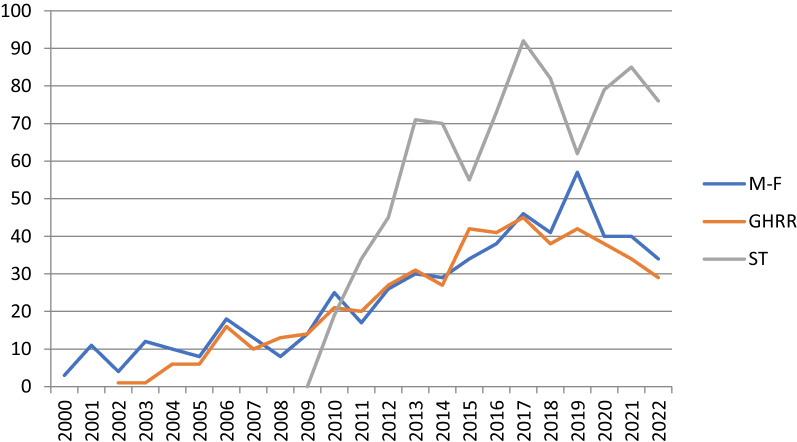
Annual output of articles citing each text, 2000–2022. Source: Scopus

We searched Scopus for work citing *Murray and Frenk*, *GHRR,* and *STHSS*, then downloaded resulting data in an Excel spreadsheet where we eliminated visible redundancies due to variations in spelling (Fig. 2).

While the number of annual articles citing *Murray and Frenk* remained stable and those of *GHRR* rose slightly during the first years of the twenty-first century, the publication of *STHSS* in 2009 as well as the republication of *GHRR* a year earlier seem to have encouraged references to all three framework publications. Citations of *STHSS* quickly outstripped those of the other publications and since 2019 have continued to rise while the two others have declined slightly. (The spike for *Murray and Frenk* in 2019 is due to an anomaly, the republication of one article in 27 different journals).

The total number of articles citing *STHSS* is considerably larger those for the other two frameworks, despite its later publication start. As mentioned earlier, the imbalance between the number of citations around each article reflects several factors. Both the scale of the projects they represent and their goals differ substantially. The Flagship program aims primarily not to produce scholarly literature but to transform systems by offering practical guidance to policy makers. Murray and Frenk was elaborated to evaluate national health systems using quantitative measures. The *STHSS* framework reflects a particularly wide set of goals and a broad group of authors seeking to study and evaluate systems as well as to change policy. It is supported by the AHPSR which directly funds systems research and special issues of journals (e.g. [21]) while its recent budgets list the number of peer reviewed articles the organization has helped to publish.

In all three lists, the overwhelmingly majority of articles are in English (over 95%). This certainly reflects the dominance of the English language in scientific publishing and may also reflect the biases of our data base. *Murray and Frenk* has the largest number of foreign language articles, mostly Spanish, which possibly reflects Frenk’s stature as a Latin American scholar-policymaker. While several well-published authors cite more than one framework article, most lean strongly toward one. For instance, Michael Reich cites *GHRR* in 24 articles and does not cite the other framework pieces; Julio Frenk cites *Murray and Frenk* in 18 papers out of 25, Lucy Gilson cites *STHSS* in 18 papers out of 22 and Kara Hanson in 6 of 7. When more than one framework is mentioned, one is frequently cited in passing or as part of a review of existing frameworks. As we shall see using more restricted sets of sources, the three publications generate quite distinct citation communities representing distinctive intellectual worlds. A similar divide is found in institutional affiliations of the corresponding authors (Table 2). Harvard is strikingly dominant in the *GHRR* dataset with over 15% of authors based at that institution. The World Bank—Harvard’s Flagship partner—comes in at a distant second at 2.3%. The *Systems Thinking* articles show a more balanced and international distribution. Here too, a few key institutions are prominent—most notably the London School of Hygiene and Tropical Medicine (LSHTM), AHPSR/WHO, and the Swiss Tropical and Public Health Institute—but they are followed closely by a wide range of institutions. While the most prominent institutions in all three sets are from high income countries, a notable proportion of authors citing *GHRR* and *Murray and Frenk* are affiliated with institutions in middle income countries like Iran and Mexico. In contrast, *STHSS* authors not based in high-income countries work frequently in African institutions, reflecting priorities at WHO and AHPSR and the close links between institutions like LSHTM and those in former British colonies on that continent. The *Murray and Frenk* citation cluster has a more geographically balanced distribution than GHRR and is the only one with authors from the University of Washington, the home of Murray and the Global Burden of Disease Project. In summary, all three sets are dominated by a handful of institutions in high income countries, but among those remaining, *STHSS* is more likely to be cited by authors in African countries while GHRR while *Murray and Frenk* are more cited by those in Mexico and Iran.

**Table 2:** Most common institutional affiliations of corresponding authors (> 1%)

Murray and Frenk articles			GHRR articles			STHSS articles		
Institution	#	%	Institution	#	%	Institution	#	%
Johns Hopkins	28	6.2	Harvard	68	15.6	LSHTM	36	5.0
Harvard	22	4.9	World Bank	10	2.3	AHPSR/WHO	23	3.2
WHO	19	4.2	National Institute of Public Health of Mexico (INSP)	9	2.1	Swiss Tropical and Public Health Institute	23	3.2
LSHTM	13	2.9	LSHTM	8	1.8	University of Toronto	15	2.1
National Institute of Public Health of Mexico (INSP)	12	2.7	AHPSR/WHO	8	1.8	University of Cape Town	14	1.9
Mexican Ministry of Health	10	2.2	Johns Hopkins	6	1.4	University of the Western Cape	14	1.9
London School of Economics	7	1.6	Tehran University of Medical Sciences	6	1.4	Johns Hopkins	13	1.8
Tehran University of Medical Sciences	7	1.6	University of Queensland	6	1.4	Columbia	11	1.5
University of Washington	7	1.6	Abt Associates	5	1.1	Harvard	10	1.4
University of York	7	1.6	Imperial College London	5	1.1	KEMRI/Wellcome Trust, Kenya	10	1.4
Shahid Beheshti University of Medical Sciences	5	1.1	Isfahan University of Medical Sciences	5	1.1	University of Lucerne	10	1.4
University of Amsterdam	5	1.1	National University of Singapore	5	1.1	University of Sydney	10	1.4
University of New South Wales	5	1.1	Shahid Beheshti Univ. of Med. Sci	5	1.1	Imperial College London	9	1.2
						Karolinska Institute, Sweden	9	1.2
						Australian National University	7	1.0
						University of Zambia	7	1.0

Source: Scopus

Moving from institutions to the countries where institutions are located adds a further dimension to our analysis (Table 3). In all three samples, the United States is the single largest contributor of corresponding authors, while the United Kingdom is always in second or third place. Canada, Australia, and Switzerland are also all in the top ten countries for each list. A large portion of Switzerland’s impact is accounted for by the WHO institutions based in Geneva. But focus on countries also illustrates the distinctiveness of each of these citation networks. For instance, after the United States and United Kingdom, Murray and Frenk’s most frequent authorial countries are Mexico and Iran. The rest of their top ten is composed of Western European countries as well as former British settler-colonies. In the case of *GHRR*, American-based authors are even more prominent, representing over a third of the sample as opposed to a fifth in the two others, with the British authors declining to third place behind Iran. While Mexico retains a place in the *GHRR* articles, it has less than half the proportional impact it had upon the *Murray and Frenk* list—while China’s proportional influence is doubled. Meanwhile, Switzerland declines to 9th place in the *GHRR* sample, reflecting the distance between the Harvard-World Bank nexus and the WHO-AHPSR world. *STHSS* citations have no contributing authors in low- and middle-income countries (LMICs) among the top five countries represented. However, it has the largest representation of citing authors from Africa (particularly South Africa and Kenya). In comparison, South Africa and Ethiopia are the only major sources of publications by authors located in Africa who cite *Murray and Frenk*, while *GHRR* gets a bit more representation from this continent. If South Africa is the major LMIC source of citations for *STHSS*, the most prominent LMICs for *Murray and Frenk* and *GHRR,* are Mexico, Iran, China, and India.

**Table 3:** Countries of corresponding authors’ institutional affiliation (1.0% of more)

Murray and Frenk articles			GHRR articles			STHSS articles		
Country	#	%	Country	#	%	Country	#	%
United States	94	20.9	United States	150	34.4	United States	129	17.9
United Kingdom	57	12.7	Iran	40	9.2	United Kingdom	101	14.0
Mexico	31	6.9	United Kingdom	35	8.0	Australia	77	10.7
Iran	24	5.3	Canada	18	4.1	Switzerland	68	9.4
Switzerland	23	5.1	Australia	17	3.9	Canada	44	6.1
Canada	16	3.6	China	15	3.4	South Africa	37	5.1
Germany	16	3.6	Germany	15	3.4	Belgium	22	3.1
Australia	12	2.7	Mexico	13	3.0	Kenya	19	2.6
Netherlands	12	2.7	Switzerland	11	2.5	Sweden	18	2.5
Spain	11	2.4	India	8	1.8	India	13	1.8
Norway	9	2.0	South Africa	8	1.8	China	11	1.5
South Africa	9	2.0	Netherlands	6	1.4	Ghana	11	1.5
Belgium	8	1.8	Singapore	6	1.4	Germany	10	1.4
India	8	1.8	South Korea	6	1.4	Ireland	10	1.4
China	7	1.6	Ghana	5	1.1	Iran	9	1.2
Italy	7	1.6	Italy	5	1.1	Norway	9	1.2
Sweden	6	1.3	Nigeria	5	1.1	Zambia	9	1.2
Turkey	6	1.3	Turkey	5	1.1	Italy	8	1.1
Ethiopia	5	1.1				Netherlands	7	1.0
						Pakistan	7	1.0
						Spain	7	1.0
						Uganda	7	1.0

Source: Scopus Metadata

Papers citing the three frameworks appear in many of the same journals, representing a narrow range of subjects around, global and public health, health policy, and health management (Table 4). *Health Policy and Planning* is the top journal for both *Murray and Frenk* (4.4%) and *STHSS* (6.0%) citations, as well as the second most frequent for *GHRR* (7.1%). All three frameworks are also cited by multiple articles in *BMC Health Systems Research*, the *Lancet*, *Social Science and Medicine*, the open access and global health formats of the *British Medical Journal, PLoS ONE,* and *International Journal of Health Planning and Management*. But there are differences. *Murray and Frenk* and *GHRR* citing articles are notably missing from *Health Research Policy and Systems*, suggesting some distance from the WHO/AHPSR world. *GHRR* citing authors publish most often in *Health Systems and Reform*, a journal edited by Michael Reich, and which began publication only in 2015. Following its inception, 13% of the *GHRR*-citing articles were published in this journal. *Health Research Policy and Systems,* which appeared almost twenty years after *Health Policy and Planning* and which publishes in collaboration with the WHO, has almost caught up with the latter journal in terms of the number of articles citing *STHSS*. Some of those citing *Murray and Frenk* publish in Latin American journals (for reasons already mentioned), whereas some of those citing *GHRR* do so in Iranian journals. The lists also display disciplinary distinctions. For instance, *GHRR* citing journals include the only one with an explicit economic focus among the three datasets—*Health Economics, Policy, and Law*. Among those citing *STHSS*, a few publish in methodological journals like *Implementation Science*—a subject which the WHO, AHPSR and other international agencies have enthusiastically taken up [52, 11]—and more neglected areas of Global Health like mental health. For the most part, however, citing articles in all three cases are published in a narrow range of specialized journals.

**Table 4:** Most cited periodicals (> 0.9%)

Murray and Frenk (*n* = 451)		GHRR articles (*n* = 436)		STHSS articles (*n* = 721)	
Periodical	# (%)	Periodical	# (%)	Periodical	# (%)
Health Policy and Planning	20 (4.4%)	Health Systems and Reform	33 (7.6%)	Health Policy and Planning	43 (6.0%)
BMC Health Services Research	19 (4.2%)	Health Policy and Planning	31 (7.1%)	Health Research Policy and Systems	42 (5.8%)
Health Policy	18 (4.0%)	International Journal of Health Planning and Management	20 (4.6%)	BMC Health Services Research	38 (5.3%)
Salud Publica de Mexico	14 (3.1%)	Lancet	17 (3.9%)	PLoS ONE	16 (2.2%)
Lancet	13 (2.9%)	BMC Health Services Research	14 (3.2%)	BMC Public Health	15 (2.1%)
BMJ Open	11 (2.4%)	Health Policy	14 (3.2%)	BMJ Open	15 (2.1%)
International Journal for Quality in Health Care	9 (2.0%)	Social Science and Medicine	9 (2.1%)	BMJ Global Health	14 (1.9%)
BMJ Global Health	7 (1.6%)	Bulletin of the WHO	7 (1.6%)	Globalization and Health	14 (1.9%)
International Journal of Health Planning and Management	7 (1.6%)	International Journal of Health Policy and Management	7 (1.6%)	Social Science and Medicine	13 (1.8%)
International Journal for Equity in Health	6 (1.3%)	BMC Public Health	6 (1.4%)	Implementation Science	11 (1.5%)
Frontiers in Public Health	6 (1.3%)	BMJ Global Health	6 (1.4%)	International Journal of Health Policy and Management	11 (1.5%)
PLoS One	6 (1.3%)	Iranian Journal of Public Health	6 (1.4%)	Health Policy	10 (1.4%)
Social Science and Medicine	6 (1.3%)	Health Affairs	5 (1.2%)	Lancet	10 (1.4%)
International Journal of Environmental Research and Public Health	5 (1.1%)	International Journal for Equity in Health	5 (1.2%)	PLoS Medicine	10 (1.4%)
International Journal of Health Policy and Management	5 (1.1%)	BMJ Open	4 (0.9%)	Global Health Action	9 (1.3%)
BMC Public Health	4 (0.9%)	Globalization and Health	4 (0.9%)	Journal of Evaluation in Clinical Practice	9 (1.3%)
European Journal of Public Health	4 (0.9%)	Health Economics, Policy and Law	4 (0.9%)	Global Public Health	8 (1.1%)
Pan American Journal of Public Health	4 (0.9%)	Journal of Isfahan Medical School	4 (0.9%)	International Journal of Environmental Research and Public Health	7 (1.0%)
		PLoS ONE	4 (0.9%)	International Journal of Mental Health Systems	7 (1.0%)

Source: Scopus Metadata

## Thematic content in most cited papers

To delve more deeply into the thematic content of the three data sets, we isolated citing articles that were themselves referenced 20 or more times. This gave us 170 articles citing *STHSS*, 107 citing *GHRR*, and 111 citing *Murray and Frenk*. In total, the sample thus includes 369 distinct articles, of which only 17 (4.6%) appear in more than one list, confirming the distinctiveness of each citation community. An interesting feature of all three collections is that while they all cite one of the framework publications, only a minority (35% in *STHSS*, 21% in *GHRR*, 24% in *Murray and Frenk*) discuss that framework in the text. And of these, the framework is mentioned in many cases only in passing, suggesting just how varied motives for citation can be.

Looking more closely at article themes (Table 5), we note that all three citation groups cover similar ground, with differences that often reflect the purpose and orientation of the original works being cited. *Murray and Frenk* was created as a framework for evaluating national health systems, a goal reflected in the outsized interest in both system-evaluation (33.3%) and program-evaluation (18.0%) articles, which together make up over half this sample, compared to roughly a third of *GHRR* and a quarter of *STHSS* articles. A strong emphasis on patient satisfaction and system responsiveness as an end goal of system reform in the original paper is reflected in the relatively greater interest in this subject among citing articles. There is simultaneously weaker interest in health system strengthening and reform than among the other citation collections. *Murray and Frenk* citations have various links to the GBD project (see below) which emphasised the global burden of noncommunicable diseases (NCDs). This might account for the interest that citing articles show in several of these conditions. This is the only citation group that discusses GBD data, albeit infrequently, which suggests that all three cited publications are more interested in systems than in diseases conditions and that GBD is not viewed as particularly relevant for systems work.

**Table 5:** Thematic content

Theme	*Murray and Frenk* (*n* = 111)	*GHRR* (*n* = 107)	*STHSS* (*n* = 170)
**Meta research**			
Systems Thinking/Perspective/Frameworks	4 (3.6%)	6 (5.6%)	49 (28.8%)
HPSR/HSR	2 (1.8%)	1 (0.9%)	12 (7.1%)
Ecohealth			4 (2.4%)
**Normative goals research**			
PHC	2 (1.8%)	6 (5.6%)	6 (3.5%)
Equity/Fairness	13 (11.7%)	16 (15.0%)	14 (8.2%)
MDGs/SDGs	3 (2.7%)	2 (1.9%)	4 (2.4%)
Social determinants of health	1 (0.9%)		5 (2.9%)
**Health policy research**			
Health Systems-Strengthening/ Reform	13 (11.7%)	27 (25.2%)	36 (21.2%)
Health Financing/Provider Payments	13 (11.7%)	42 (39.3%)	15 (8.8%)
Global Burden of Disease	3 (2.7%)		
Health System Resilience/Crisis Response	1 (0.9%)	2 (1.9%)	6 (3.5%)
Health Governance/Stewardship	10 (9.0%)	7 (6.5%)	20 (11.8%)
Evidence-Informed Policies/Knowledge Translation	3 (2.7%)	3 (2.8%)	18 (10.6%)
Health Policy Making Processes	1 (0.9%)	11 (10.3%)	8 (4.7%)
Health System Assessments	37 (33.3%)	17 (15.9%)	17 (10.0%)
Program Evaluations	20 (18.0%)	16 (15.0%)	27 (15.9%)
Scaling up programs		1 (0.9%)	8 (4.7%)
Global social policy		2 (1.9%)	1 (0.6%)
**Health admin research**			
Health Workers/Human Resources	7 (6.3%)	3 (2.8%)	14 (8.2%)
Supply Chain Management		2 (1.9%)	1 (0.6%)
Community health			3 (1.8%)
Health information systems	1 (0.9%)		3 (1.8%)
Patient Satisfaction/System Responsiveness	17 (15.3%)	5 (4.7%)	1 (0.6%)
Decentralization		2 (1.9%)	
**Health problems research**			
Tropical Diseases	10 (9.0%)	5 (4.7%)	28 (16.5%)
Noncommunicable Diseases (NCD)	13 (11.7%)	3 (2.8%)	12 (7.1%)
Malnutrition			2 (1.2%)
Child and maternal health	5 (4.5%)	4 (3.7%)	24 (14.1%)
Post-conflict/conflict health	1 (0.9%)	2 (1.9%)	2 (1.2%)
Pharmacological therapies	2 (1.8%)	3 (2.8%)	11 (6.5%)
Non-pharma therapies	6 (5.4%)	3 (2.8%)	8 (4.7%)

*GHRR* was promoted as a program to teach procedures to facilitate health system reform (reflected in the title of the journal most closely associated with it), so it is hardly surprising that the citing articles show particular interest in issues of health reform (25.2%) and the policy-making process (10.3%). The focus on financing and payment (39.3%) is also unsurprising, given the economics background of several *GHRR* authors and the two financial “control knobs” in the framework. It is worth noting that among the different subfields of financing, this citation grouping has the highest proportion of articles dealing with universal healthcare (6.5%) and is the only cluster to discuss catastrophic expenditures (8.4%). It also shows the highest proportion of articles dealing with issues of equity (15%) and Primary Health Care (5.6%), somewhat surprising in a cluster built around an initiative co-sponsored by the World Bank.

*STHSS* was about developing methodologies for an applied field devoted to health systems research, which accounts for the interest of citing articles in system thinking and frameworks (28.8%) and other health system research methodologies (7.1%.). This perspective also accounts for the relatively high proportion of articles discussing evidence-informed research in policymaking (10.6%). *STHSS* citing articles show somewhat greater but still limited interest than those in other citation communities in specific health problems, particularly tropical diseases and child and maternal health. They show less interest in overall health system performance and more in program assessment. This likely reflects their focus on low-income countries with huge problems, limited capacity for generating data, and a plethora of programs to deal with these problems. Attention to the latter may also explain their stronger interest in finding ways to scale up programs. Conversely, the greater representation of NCDs (11.7%) among *Murray and Frenk* citing articles may reflects their greater attention to high and middle-income countries and somewhat closer proximity to the Global Burden of Disease Project. In other respects, the three citation groups show few major differences. Social determinates of health are largely neglected in all three. Differences among them in the proportion of articles about health administration issues are small.

## Discussion

This study of three citation communities based on system frameworks confirms the well-recognized insight that authors can have many reasons for citing an article, aside from using it for data or to back up arguments (which only a minority of articles do). More originally, the clearest lesson of this exercise is how divided into distinctive silos these citation networks appear to be even though they all publish in the same narrow range of specialized journals. This confirms other work [50] that suggests that Global Health literature more generally is similarly fragmented and heterogenous. Each of the three most popular system frameworks attracts a distinct citing community that reflects questions it was designed to operationalize, patterns of institutional affiliation and authority, and the stature of individuals associated with it. Based on this data, it is impossible to tell if these aggregations are fleeting or reflect more permanent communities. But the clear differences among them with respect to publishing journals and institutional affiliations suggest that they may well be parts of more permanent expert networks shaped by their supporting infrastructures, the concerns that they represent, or personal links (in the case of *Murray and Frenk*, see below). This hypothesis will of course need to be tested by further research. All three citation groupings are dominated by authors from the Global North, but authors from LMIC are nonetheless active. Among the latter, those referencing *GHRR* and *Murray and Frenk* come mainly from a handful of middle-income countries—most notably Mexico, Iran, and to a lesser extent China—while those citing *STHSS* tend to come from a broader range of low- and middle-income countries particularly in Africa.

As the official framework of the WHO and several other agencies, *STHSS* has gradually become the most referenced framework publication. But *GHRR* has also spread widely thanks in part to its prestigious Harvard/World Bank provenance and its popular teaching program. Since *Murray and Frenk* is not actively promoted by an institution, citations seem linked less by infrastructures than by personal ties to the world of GBD and to the two co-authors, as well as ongoing interest in evaluating health systems. Of the 14 authors with 3 or more citations of this articles (excluding Murray and Frenk themselves), two are closely connected to Frenk through co-authorship, expertise in Mexico and, in one case, marriage (Gomez-Dantes and Knaul). Three have co-authored articles with Murray (Valentine, Gakidu, and Agyepong) with two of these having worked with him at WHO or the Institute for Health Metrics and Evaluation. Two others (Achoki and Crews) have published articles based on GBD data. The remaining authors have written about burdens of specific diseases, performance evaluation, and Mexico (Puentes Rosas, Feller, Forouzan, Papanicolas, Veillard, Verguet). Only one (Blanchet) has no clearly visible connection to the authors or their themes.

Another way of getting at personal connections is by looking at the corresponding authors that cite one framework at least three time and another at least twice. (Secondary authorship is more common but less compelling because of the tendency to collect many co-authors, with some contributing little besides their names.) Of the 10 authors who fit these criteria, 5 cite both *Murray* and *Frenk* and *GHRR*. Since Reich, Frenk and Murray have all worked there at one time or another, it is hardly surprising that of these five, temporary or long-term Harvard affiliation looms large (Verguet, Knaul, Gakidou, and Gómez-Dantés, MPH 1991).

## Conclusion

While this paper has illustrated some of what can be learned by analyzing the large citation communities that certain articles are able to generate, it worth noting its limitations. It raises intriguing questions that it cannot provide definitive answers to and that must be left for future research. We cannot really tell whether these communities are fleeting or whether, as we suspect, they are more permanently held together by institutional infrastructures and personal connections. Similarly, most of our explanations of specific characteristics and patterns are not based on the data shown but are rather speculative interpretations based on years of reading the HPSR literature. They will need to be verified using very different research approaches. A more comprehensive scientometric analysis of the HPSR literature using more sophisticated correlational mapping techniques is currently underway and should allow us to answer at least some of these questions. These may or may not tell us something more about the three citation groups we have been examining but will certainly advance our understanding of HPSR as an aggregation of communities and networks.

## Data Availability

The datasets during and/or analysed during the current study available from the corresponding author on reasonable request.

## Footnotes

1 The WHO and European Observatory on Health Systems and Policies recently produced a framework to conceptualize the assessment of health system performance with the goal of harmonizing such assessments [54].
